# Frequency of Normal Birth Length and Its Determinants: A Cross-Sectional Study in Newborns

**DOI:** 10.7759/cureus.10556

**Published:** 2020-09-20

**Authors:** Saira Jamshed, Farah Khan, Sarwat Khalid Chohan, Zakia Bano, Shizra Shahnawaz, Adnan Anwar, Atif A Hashmi

**Affiliations:** 1 Obstetrics and Gynecology, Hamdard University Hospital, Karachi, PAK; 2 Obstetrics and Gynecology, Sobraj Maternity Hospital, Karachi, PAK; 3 Obstetrics and Gynecology, Dar-Ul-Sehat Hospital, Karachi, PAK; 4 Obstetrics and Gynecology, Jinnah Postgraduate Medical Center, Karachi, PAK; 5 Physiology, Al-Tibri Medical College, Karachi, PAK; 6 Stereotactic Radiosurgery/Radiation Oncology, Al-Tibri Medical College, Karachi, PAK; 7 Pathology, Liaquat National Hospital and Medical College, Karachi, PAK

**Keywords:** mothers, infant, newborn, association, cross sectional study, normal birth length

## Abstract

Objectives

There are several factors that may affect the length and height of the infant. Maternal factors include a wide array of factors (anthropometric, hematological, or genetic), which can affect newborn health determinants. The objective of this study was to evaluate the frequency of normal birth length and its determinants in newborns.

Methods

This retrospective cross-sectional study was carried out at the Obstetrics and Gynaecological Department of Hamdard Hospital, Karachi, Pakistan, from March 1, 2019, to August 31, 2019. The relevant data were gathered by trained data collectors with the help of a structured questionnaire designed specifically for the study after taking written informed consent from all the participants. Data analysis was performed using Statistical Package for Social Sciences Version 20. Binary logistic regression was applied to develop a risk assessment model for the study outcome.

Results

Out of 195 pregnant mothers, 57 (29.2%) had low birth weight infants. Mean age of mothers was 29.29±5.22 years, 142 (72.8%) had BMI of 25.0 or more, 102 (52.3%) had hemoglobin between 10 to 11 mg/dL, 172 (88.2%) used to take vitamin C and iron during pregnancy, 136 (69.7%) consumed extra meals during pregnancy, and only 5 (2.6%) were tobacco smokers/chewers. Hundred (51.3%) newborns had normal birth length, i.e., >48 cm. The mother's mid-arm circumference > 22 cm (adjusted odds ratio [AOR]: 4.719; 95% CI: 2.337-9.527; p<0.001), consumption of extra meals during pregnancy (AOR: 3.947; 95% CI: 1.627-9.574; p=0.002), hemoglobin > 11 mg/dL (AOR: 4.314; 95% CI: 1.779-10.463; p=0.001), and adequate rest during pregnancy (AOR: 3.798; 95% CI: 1.464-9.848; p=0.006) were significantly associated with normal birth length of the infants, i.e., >48 cm.

Conclusions

Mother's mid-arm circumference > 22 cm, consumption of extra meals during pregnancy, hemoglobin > 11 mg/dL, and adequate rest during pregnancy were found to be significant predictors of normal birth length of the infants.

## Introduction

The average normal birth length is defined as the full-term length of a newborn measuring 19-20 inches or 49-50 centimeters. However, a length of around 18.5-20.9 inches or 47-53 centimeters is also considered as normal birth length [1]. It has been observed after measuring the baby’s length from head to foot that the birth length of male babies is slightly more than female babies [2]. Alterations in size or length of a baby are referred to as small size for gestational age (SGA), when infants are less than 10th percentile of babies of the same gestational age, and as large size for gestational age (LGA), when they are more than 90th percentile of same gestational age babies [3].

The terms SGA and LGA refer to the lower and higher weight, height, and head circumference of the infant’s body as compared to the expected length and weight, respectively [4]. Babies born with SGA or LGA are approximately 20% of all the normal births [5]. Both types of children born either with SGA or LGA are associated with a greater risk of morbidity and mortality at the time of birth. However, they may show realignment in their growth, which totally depends on their genetic growth potential [6].

The specific growth patterns for growth realignment in infants born with SGA and LGA are still not much known. However, different studies revealed several associated factors such as the infants born with LGA have a higher potential to be obese in their later life [7]. Furthermore, it may cause prolonged delivery, excessive maternal hemorrhage, genital tract tears, and higher risk of cesarean sections in mothers [8]. Due to the risk of LGA evaluated from ultrasound, intrauterine growth inhibitors are usually given, which may increase the risk of fetal death within the uterus of the mother. Furthermore, it may cause severe intestinal infection, hypoxia during delivery, diabetes, cardiovascular disease, and hypoglycemia in neonates [9]. It is because any changes in the uterus can badly affect the health of the baby in later life [10].

There are several factors that may affect the length and height of the infant. In this way, genetics play a major role as infants are more likely to be of similar height to their parents. However, it is not always apparent and a tall adult may reveal a shorter length at birth and in the first two years of his life [11]. Other than genetics, maternal nutrient intake is also considered as an important parameter that contributes to birth weight and length of a child. However, the direct association between diet quality of mother and its effect on birth length of a child has not been clearly verified [12]. Moreover, in recent years, the analysis of dietary pattern has been conducted, which revealed that unhealthy dietary patterns may increase the chances of SGA in babies [13].

Another factor includes the evaluation of mid-arm circumference. Kanawati and McLaren were the first authors who proposed the mid-arm circumference/head circumference (MAC/HC) ratio, which can be measured by using non-expensive equipment and with simple training [14]. Evaluation of mid-arm circumference is strongly linked to the birth weight and length of a baby and is considered as a suitable indicator for the evaluation of insufficient weight and height. However, it does not conducted as a part of routine evaluation for newborns [14].

Another factor that affects the baby’s health and physical appearance is maternal rest during pregnancy. It has been suggested that due to maternal stress or less rest during pregnancy, babies are physically affected. Moreover, the development of the offspring can be affected both positively and negatively depending upon the maternal energy level [15]. Moreover, it has also been reported that less maternal energy and high stress level with low rest during pregnancy result in sub-optimal growth of a baby at birth and even during different stages of infancy [16]. Also, during pregnancy, mothers are at high risk of sleep deprivation due to several reasons such as hormonal changes, mood, body temperature, and so on. This deprivation in sleep causes high stress and depression, which ultimately increase the risk of adverse fetal growth [16].

Therefore, the aim of this study is to evaluate the frequency of normal birth length and different associated factors that affect the length and growth in newborns such as maternal BMI, mid-arm circumference, consumption of extra meals, level of hemoglobin, and adequate rest during pregnancy.

## Materials and methods

This retrospective cross-sectional study was carried out at the Obstetrics and Gynaecological Department of Hamdard Hospital, Karachi, Pakistan, from March 1, 2019, to August 31, 2019. A total of 195 healthy mothers and their newborns were included in the study. Institutional Review Board of the hospital gave the ethical approval of the study. Written informed consent was taken from all of the participants prior to their inclusion in the study.

The percentage frequency of the study outcome was taken as 50% for the most liberal estimate, with 95% confidence level and 7% precision, and the minimum sample size was calculated to be 196 participants.

Healthy pregnant females with either singleton or multiple pregnancies with at least three antenatal checkups were included, whereas females giving birth at gestational age less than 34 weeks or above 41 weeks and six days or with a complicated pregnancy were excluded from the study. All the pregnancies were followed up to the delivery. All the relevant data were gathered by trained data collectors with the help of a structured questionnaire, and the weight and height/length of the mothers and children were also measured.

Data were analyzed using Statistical Package for Social Sciences Version 20.0 (IBM Corp., Armonk, NY, USA). Descriptive analysis was performed by generating means and standard deviations for continuous variables, and frequencies and percentages for categorical variables. Binary logistic regression was applied to develop a risk assessment model for the study outcome while keeping the significance level at 0.05.

## Results

There were a total of 195 pregnant females selected for the study. Frequency of low birth weight infants was found to be 57 (29.2%) in these women. The mean age of the mothers was 29.29±5.22 years; 142 (72.8%) of them had BMI of 25.0 or more, 102 (52.3%) had hemoglobin between 10 to 11 mg/dL, 172 (88.2%) used to take each of vitamin C and iron during pregnancy, 136 (69.7%) consumed extra meals during pregnancy, and only 5 (2.6%) were tobacco smokers/chewers (Table 1).

**Table 1:** Participants' profile

Variables (n=195)	Mean±SD/Frequency (%)
Birth weight (kg)	
Up to 2.5	57 (29.2)
More than 2.5	138 (70.8)
Maternal age (years)	29.29±5.22
Maternal BMI before pregnancy	
Up to 24.9	53 (27.2)
25.0 or more	142 (72.8)
Maternal hemoglobin (mg/dL)	
<10	44 (22.6)
10 to 11	102 (52.3)
>11	49 (25.1)
Vitamin C intake	
Yes	172 (88.2)
No	23 (11.8)
Iron intake	
Yes	172 (88.2)
No	23 (11.8)
Extra meals consumed	
Yes	136 (69.7)
No	59 (30.3)
Tobacco smoking/chewing	
Yes	5 (2.6)
No	190 (97.4)

Hundred (51.3%) of the newborns had normal birth length, i.e., >48 cm.

The study results further showed that mid-arm circumference > 22 cm (adjusted odd ratio [AOR]: 4.719; 95% CI: 2.337-9.527; p<0.001), consumption of extra meals during pregnancy (AOR: 3.947; 95% CI: 1.627-9.574; p=0.002), hemoglobin > 11 mg/dL (AOR: 4.314; 95% CI: 1.779-10.463; p=0.001), and adequate rest during pregnancy (AOR: 3.798; 95% CI: 1.464-9.848; p=0.006) were significantly associated with normal birth length of the children, i.e., > 48 cm, whereas mothers who had mid-arm circumference > 22 cm, consumed extra meals during pregnancy, had hemoglobin >11 mg/dL, or took adequate rest during pregnancy had significantly higher odds of having a child with normal birth length than those who did not (Table 2).

**Table 2:** AORs of associations between maternal factors and birth length of the newborns AOR, adjusted odds ratio

Variables (n=195)		AOR	95% CI		p-Value
			Lower	Upper	
Body mass index	25.0 or more	1.832	0.878	3.825	0.107
Mid-arm circumference	>22 cm	4.719	2.337	9.527	<0.001
Extra meals	Consumed	3.947	1.627	9.574	0.002
Hemoglobin	>11 mg/dL	4.314	1.779	10.463	0.001
Adequate rest during pregnancy	Taken	3.798	1.464	9.848	0.006

## Discussion

This study was aimed at evaluating the frequency of normal birth length and its determinants in newborns. It was found that 51.3% of newborns had a normal birth length, i.e., >48 cm. The results further revealed mid-arm circumference > 22 cm, consumption of extra meals during pregnancy, hemoglobin > 11 mg/dL, and adequate rest during pregnancy to be significantly associated with normal birth length of the newborns.

The major parameter in mother that affects the fetal growth is the hemoglobin level. During pregnancy, it has been reported that risk of anemia is increased to more than fourfold from the first to third trimester [17]. Anemia is the major health problem that arises during pregnancy and affects approximately 50% of pregnant women, and the major adverse outcome of maternal anemia is the risk of perinatal mortality, premature delivery, and low birth weight and height of an infant. However, it is a fact that the hemoglobin level certainly drops in the middle of trimester [18]. According to the report published by World Health Organization, during the first and third trimesters, women with hemoglobin levels less than 11 g/dL and during second trimester and those with levels less than 10.5 g/dL are considered anemic [19]. The presence of anemia in pregnant women may likely to affect the growth of the fetus. Furthermore, low birth weight/growth and pre-mature delivery have been reported to be directly associated with anemia in pregnancy [20]. Similar findings were reported in our study as well in which hemoglobin played a significant role in determining the birth length of the newborns.

Other than hemoglobin, maternal obesity has also been observed to play a substantial role in delivering infants with high birth weight. The association between adult weight and birth weight might have a correlation. To study this, a research based on the association of maternal obesity to neonatal birth weight found that among 162,676 mothers that delivered infants, the odd ratio of delivering infants with high birth weight was 1.50 in overweight mother, 1.77 among obesity class I mothers, 2.77 among obesity class II mothers, and 2.04 in mothers with class III obesity. Mothers who delivered babies that were LGA were observed to have a higher BMI [21].

In yet another study, high birth weight was significantly associated with maternal obesity (p<0.05). The study concluded that the infants of overweight mothers were reported to have greater body fat mass as compared with the infants of lean mothers, which suggests that maternal obesity might be a predisposing factor for fetal metabolism to favor fat storage [22]. In contrast, our study findings did not report a significant association of maternal obesity with their infant’s birth weight. Possible reasons may be limited sample size and most mothers being below the obesity ranges of BMI.

A study determined to find out the possible reasons for mothers affecting newborns’ anthropometric measurements reported that a substantial association was observed between maternal anthropometry and neonatal anthropometry. Maternal height, birth order, BMI, eating extra meals, taking adequate rest during pregnancy, maternal hemoglobin, and mid-arm circumference were all found to be significantly linked to newborn’s birth weight. Likewise, a positive history of iron or multivitamin intake, folic acid, and vitamin C, and negative history of smoking were seen to have positive effects on newborn’s birth length as well as overall health [23]. Similarly, in our study as well, a significant association of hemoglobin, mid-arm circumference, taking extra meals during pregnancy, and adequate rest with birth length was observed.

In addition to the aforementioned maternal factors affecting birth length of newborns, studies have reported that maternal blood pressure throughout pregnancy, hypertension, coronary or any other cardiovascular disease status, and insulin resistance in diabetic mothers also tend to have substantial effects on the infants’ health [24]. Genetic factors also tend to play an important role along with intra-uterine environment and are pivotal in explaining fetal growth and affecting birth length [25].

Limitations

A small sample size and non-probability sampling method are acknowledged to be the prime limitations of the study.

## Conclusions

Mid-arm circumference > 22 cm, consumption of extra meals, hemoglobin > 11 mg/dL, and adequate rest during pregnancy were found to be significant predictors of normal birth length of the children. Careful evaluation of the factors that need assessment during antenatal visits is recommended in order to have a better chance of having newborns with normal birth length.
